# Age is a prognostic factor affecting survival in lung cancer patients

**DOI:** 10.3892/ol.2013.1566

**Published:** 2013-09-06

**Authors:** FARUK TAS, RUMEYSA CIFTCI, LEYLA KILIC, SENEM KARABULUT

**Affiliations:** Institute of Oncology, University of Istanbul, Capa, Istanbul 34390, Turkey

**Keywords:** lung cancer, elderly, prognostic factor, survival

## Abstract

Despite all efforts at management, prognosis of advanced lung cancer is extremely poor, with a median survival time of ~1 year. The number of cancer patients aged >70 years is significantly increased among the cancer patient population. The aim of this study was to investigate the clinical importance of age in lung cancer. Data from 110 patients with histologically confirmed lung cancer, who were treated and followed up in the Institute of Oncology, University of Istanbul, were recorded from medical charts. There were 100 (91%) males with a median age of 59 years (range, 35–88 years). The majority of patients had non-small cell lung cancer (NSCLC; 84%) and metastatic stage (56%). The rate of positive response to chemotherapy was lower in elderly patients (P=0.01) and the incidence of anemia was higher compared with that in younger patients (P=0.02). The majority of mortalities occurred in elderly patients (P=0.01). The median survival time of elderly patients was significantly lower compared with that of younger patients (37.8 vs. 57 weeks; P=0.009). The 1-year survival rates in younger and elderly patients were 67.3 and 42.5%, respectively. In multivariate analysis, elderly patients also had significantly poorer survival (P=0.023). In the group of elderly patients, analyses revealed that significant prognostic factors, including stage of disease and serum lactate dehydrogenase (LDH) levels, were associated with survival. Elderly patients diagnosed with small cell lung cancer had a poorer outcome compared with those with NSCLC (P=0.009), and older patients with elevated serum LDH levels had a shorter survival time compared with those with normal levels (P=0.042). In conclusion, age is one of the major prognostic factors affecting survival in lung cancer patients; therefore, patients should be managed according to age in clinical practice.

## Introduction

Currently, lung cancer is a major health problem. In the USA, an estimated 222,500 new cases of lung cancer were diagnosed and 157,300 mortalities occurred due to the disease in 2010 (1). Lung cancer is the most common fatal cancer in males and females, and is predicted to account for 29% (86,220) of all male and 26% (71,080) of all female cancer-related mortalities (1). It surpassed breast cancer as the leading cause of cancer-related mortality in females in 1987. Additionally, data indicate that it is the second most common cancer in males and females.

The prognosis of lung cancer remains poor. Up to 85% of patients are diagnosed at an advanced stage. Despite all efforts at management, prognosis of lung cancer patients is unsatisfactory, with a median survival of ~1 year and a 5-year survival rate of only 16% (1). Non-surgical treatment options, including chemotherapy or targeted therapy, have been investigated to determine whether they prolong the overall survival of patients with advanced lung cancer. Due to the moderate progress provided by chemotherapeutics, studies have investigated whether subgroups of patients may be identified to determine who would benefit most from specific treatment strategies (2). This may lead to improvement in the selection of patients with poor prognosis to be treated only with supportive care, and may avoid unnecessary adverse effects and complications of systemic chemotherapy. Certain prognostic factors are predictive of survival in patients with lung cancer. Good prognostic factors include early stage disease at diagnosis, good performance status (PS), no significant weight loss (not >5%) and female gender (2). Mutations of the tumor suppressor gene p53, the activation of proto-oncogene K-ras and other biological markers may have significant value in predicting a poor prognosis (3,4). National Comprehensive Cancer Network guidelines (2011 version) state that the age of patients in addition to the histological subtype has prognostic significance.

Cancer is one of the five leading causes of mortality in all age groups among males and females; it is the leading cause of mortality among men and women aged 40–79 years (1). Among males aged ≥40 years, cancer of the lung is the most common fatal cancer. However, among females, lung cancer ranks first in individuals aged ≥60 years. Therefore, lung cancer is accepted as a disease of the older population (5,6). The incidence of lung cancer increases with age, with 60% of patients being over the age of 65. As the geriatric population increases in the world, lung cancer continues to be an important public health issue today and in the future.

Age has been recognized as a prognostic factor in multiple cancers treated with definitive intent. In addition to numerous variables that have been reported to be significant prognostic factors in lung cancer, several studies have demonstrated that age is an important independent prognostic factor affecting survival of patients (5–7). Moreover, elderly patients usually benefit from single and combination chemotherapy regimens. Data on elderly lung cancer patients and the importance of age on survival of lung cancer patients are limited. In the present study, we aimed to identify and evaluate the known clinicopathological factors and to elucidate the clinical significance of patient age on the outcome of lung cancer.

## Patients and methods

### Patients

This study included 110 consecutive patients admitted to the Institute of Oncology, University of Istanbul (Turkey) with histologically or cytologically confirmed non-small cell lung cancer (NSCLC) and small cell lung cancer (SCLC) who were treated and followed up in our clinic; data were recorded from medical charts. Patients with bidimensional measurable disease without a history of chemo- or radiotherapy in the last six months were included in the study. The staging of metastatic patients was performed using various imaging modalities, including computed tomography (CT), magnetic resonance imaging and positron emission tomography/CT. The pathological diagnosis of lung cancer was established in accordance with the revised World Health Organization classification of lung tumors (8) and was staged according to the revised TNM staging for lung cancer (9). This study was approved by the ethics committee of the Institute of Oncology, University of Istanbul. Written informed consent was obtained from the patients.

### Treatment

The pretreatment evaluation included detailed clinical history and physical examination with a series of biochemical tests, and complete blood cell counts. Those with Eastern Cooperative Oncology Group PS ≤2 and appropriate blood chemistry tests received chemotherapy on an outpatient basis comprising platinum compounds with or without radiotherapy, depending on the stage of disease. Patients were treated with various chemotherapy regimens as a single agent or combination therapy. Regimens of single or combination chemotherapy were selected based on the PS of patients and extension of disease. Drug schemes consisted of platinum agents in combination with newer cytotoxic drugs, including paclitaxel, docetaxel, gemcitabine and vinorelbine.

Response to chemotherapy was evaluated radiologically following two to three cycles of chemotherapy according to international criteria, the revised Response Evaluation Criteria in Solid Tumors guidelines (version 1.1). Non-responders to chemotherapy and relapsed patients were treated with second-line chemotherapy if they had a good PS. Chemotherapy was continued until disease progression or unacceptable toxicity. Follow-up programs consisted of clinical, laboratory and radiological assessments performed at 8-week intervals during chemotherapy or every 12 weeks for patients receiving no anticancer treatment.

### Statistical analysis

Continuous variables were categorized using median values as the cut-off point. Assessment of correlations and comparisons between various clinical/laboratory parameters was performed using Mann-Whitney U test and Kruskal-Wallis test for 2 and 3 groups, respectively. Survival was calculated from the date of first admission to hospital to mortality resulting from any cause or to last contact with the patient or any family member. The Kaplan-Meier method was used for estimation of survival distribution and differences in survival were assessed by the log-rank statistic. Multivariate survival analysis was performed using Cox’s proportional hazards regression model. P<0.05 was considered to indicate a statistically significant difference. Statistical analysis was performed using SPSS 16.0 software (SPSS Inc., Chicago, IL, USA).

## Results

Demographic, laboratory and clinicopathological features of patients are listed in Table I. In this retrospective study, we analyzed the outcome of 110 patients with lung cancer who were treated and followed up in our clinic. There were 100 (91%) male patients with a median age of 59 years (range 35–88 years). The majority of the patients had NSCLC (84%), metastatic stage (56%) and had normal serum hemoglobin (71%), white blood cell (WBC; 77%), platelet (65%) and lactate dehydrogenase (LDH) levels (75%). Moreover, response rate to chemotherapy was almost 50%.

The distribution of prognostic factors according to the age of patients was generally similar (Table I). However, the percentage of patients who responded to chemotherapy was lower in elderly patients (37 vs. 68%; P=0.01). Similarly, the percentage of patients with anemia was higher in elderly patients compared with younger patients (39 vs. 19%; P=0.02). The majority of the mortalities occurred in elderly patients (40 vs. 17%; P=0.01).

**Figure 1:**
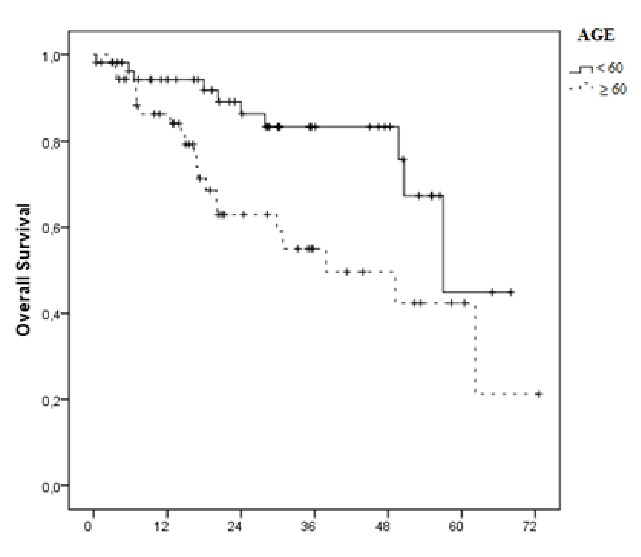
Overall survival of patients with lung cancer according to age of patients (P=0.009).

The median follow-up time was 20.3 weeks (range, 4.4–72.6 weeks). At the time of analysis, 31 patients (19.4%) had succumbed due to disease-related or unrelated factors. The median survival time of patients was 57 weeks (95% CI, 45.6–68.3 weeks). The 1-year survival rate was 55.1±7.7%. In the subset analysis, it was identified that 40% of elderly and 17% of younger patients succumbed to the disease. The median survival time of elderly patients (37.8 weeks; 95% CI, 11.6–64.0) was significantly lower than that of younger patients (57 weeks; 95% CI, 45.8–68.1) (P=0.009). The 1-year survival rates in younger and elderly patients were 67.3±11.3 and 42.5±1.0%, respectively (Fig. 1).

In the univariate analyses, elderly patients had poorer outcomes compared with younger patients (P=0.009; Table II). Additionally, higher LDH levels (P=0.001) and lack of response to chemotherapy (P<0.001) were associated with shorter survival time. In the multivariate analysis, elderly patients had a poorer prognosis (P=0.023).

Table III summarizes the analysis of the association between age of patients and various clinical and laboratory parameters. In the younger group of patients, analyses demonstrated that the significant prognostic factors associated with survival were stage of disease and serum LDH levels. Elderly patients diagnosed with SCLC had a poorer outcome compared with those with NSCLC (P=0.009) and older patients with elevated serum LDH levels had a shorter survival time compared with those with normal values (P=0.042).

## Discussion

Due to increasing life expectancy and the increased risk of cancer with aging, lung cancer is common in elderly individuals. More than half of lung cancer cases are diagnosed in patients aged >65 years (5–7). The median age at diagnosis of lung cancer is between 64 and 70 years (6,7). Age at diagnosis also depends on the health policy of the country. If elderly patients do not receive diagnostic procedures in cases of suspected lung cancer, the median age is likely to be lower than in a country where elderly patients are thoroughly explored. In a community hospital-based survey in France, patients aged ≥70 years with pathologically confirmed lung cancer represented 32% of the 5,667 patients recorded during the year 2000 and patients older than 80 years represented 18.1% (10). Conversely, in the US, patients aged >65 years represented two-thirds of the total number of lung cancer cases and the median age at diagnosis was almost 70 years, with a similar percentage of patients aged ≥80 years as in the French study (11). NSCLCs represent 85% of all lung cancer cases in the elderly, as in their younger counterparts (6). Squamous cell carcinomas are more frequent in elderly compared with in younger patients (5,6). The distribution by gender in the elderly is similar to that in younger patients in the US; whereas, in France, there is a decrease in the male/female ratio with increasing age (10,11).

Treatment of elderly patients with lung cancer poses a huge challenge (5). Although elderly patients represent the majority of lung cancer cases, they are under-represented in clinical cancer treatment trials, where they represent only 30–40% of enrolled patients (12). In National Cancer Institute-sponsored clinical trials, <1% of adults aged 75–79 years are enrolled (13). Therefore, patients in this age range may not be optimally treated (13,14). The possible explanations of this situation are the presence of comorbidities of patients, limited expectations for long-term benefit of chemotherapy and for these reasons, the exclusion of older individuals based on non-eligibility criteria.

Elderly patients included in clinical trials in which younger patients are also included, represent a highly selective population of patients, as they must satisfy extremely strict inclusion criteria. Thus, the results obtained cannot be extrapolated to the whole population of elderly patients (5,6,14–16). Therefore, recommendations for chemotherapy in elderly NSCLC patients are based on subgroup analyses of elderly patients included in clinical trials with no upper age limit, with only rare specific studies and phase trials specifically contained to elderly patients with advanced lung cancer (6,14,15,17). Only a minority of patients >65 years receive chemotherapy for an advanced NSCLC, as shown in a survey from the Surveillance Epidemiology and End Results program; only 25.8% of the 21,285 patients aged ≥66 years with NSCLC diagnosed between 1997 and 2002 (7,18).

Studies performed on elderly patients with various malignancies show that elderly patients benefit from chemotherapy to a similar extent as younger patients, with manageable side-effects (19–21). The vast majority of these subgroup analyses demonstrated that the efficacy results are similar in patients aged <70 years old and in those aged ≥70 years (20–24). In these trials, no difference in survival was observed according to age. However, there were more side-effects, including leukopenia, weight loss and respiratory and cardiovascular comorbidities, in elderly patients. The only study showing a shorter survival in elderly patients is the combined analysis of two Southwest Oncology Group trials, one comparing cisplatin alone to cisplatin-vinorelbine and the other comparing cisplatin-vinorelbine to carboplatin-paclitaxel (25–27). Severe neutropenias were more frequent in the older group; however, the occurrence of febrile neutropenia was the same.

Commonly used guidelines of oncology [American Society of Clinical Oncology (ASCO) and European Organization for Research and Treatment of Cancer (EORTC)] are based on subgroup analyses of elderly patients included in non-elderly dedicated trials, which is why specific studies dedicated to elderly patients should be conducted and elderly patients should be interpreted differently in clinical practice. Notably, several phase III trials specifically dedicated to elderly patients with advanced NSCLC have been performed (28–30). In these trials, older patients with advanced NSCLC were randomized between single-agent chemotherapy, such as vinorelbine alone, and best supportive care (28). Additionally, vinorelbine-gemcitabine treatment has been compared with vinorelbine alone and/or gemcitabine alone in two trials (29,30). As a consequence of these trials, in 2004, the ASCO recommended treating elderly patients with advanced NSCLC with a single-agent chemotherapy (31). Vinorelbine and gemcitabine were the most frequently studied agents with similar outcomes. However, single-agent docetaxel chemotherapy was found to be as effective as that with vinorelbine alone (32). Since the beginning of the last decade, single agent chemotherapy has been accepted as a standard care of NSCLC, with vinorelbine and gemcitabine being the most frequently used drugs in Europe and US (29,30,33,34), and docetaxel being the most frequently used in Japan (32,35). Platinum-based doublets have been shown to be superior to monotherapy in young and fit patients with advanced NSCLC (22,36–39). Although there were a number of indications from subgroup analyses of clinical trials that were not specifically dedicated to elderly patients, a platinum-based doublet may also benefit older patients.

Age should not be criteria of choice for the best treatment due to the ASCO recommendations in 2009 (40). Conversely, EORTC recommendations published in 2010 (41) continue to recommended monotherapy as the treatment for elderly patients with advanced NSCLC. As platinum-based doublets have been shown to be superior to monotherapy in young and fit patients with advanced NSCLC, the paradigm of treatment in elderly patients should perhaps be modified from a single agent to doublet chemotherapy. The trends of monotherapy in elderly patients aged ≥70 years with a good PS should possibly be modified favoring the combination of carboplatin with weekly paclitaxel. Whether other carboplatin-based doublets provide the same benefit remains to be evaluated

Moreover, there have been no specific trials concerned with salvage chemotherapy for elderly NSCLC patients. The only available study is a subgroup analysis of 86 patients aged ≥70 years among the total 571 patients included in a phase III study comparing pemetrexed and docetaxel as a second-line therapy (42). Outcomes of elderly patients were similar to those of younger patients and pemetrexed had a better safety profile than docetaxel in elderly patients.

Toxic events with chemotherapy are more common in elderly patients. Alterations of physiological functions, particularly renal and hematopoietic functions, with aging may explain increased chemotherapy toxicity in elderly patients (6,43–45). Drugs with renal metabolism or a high level of hematotoxicity should have their dosage correctly adjusted. Although hematological toxicity is anticipated to be more severe in elderly patients, growth factors appear to have the same efficacy as in younger patients and should be considered (46,47). Primary prophylaxis with granulocyte colony-stimulating factor (G-CSF) is of particular interest, as the highest incidence of toxicities that result in patient mortality occur during the first cycle. Prophylactic use of G-CSF should be reduced to decrease the risk of neutropenia in elderly patients with advanced NSCLC when they are treated with myelotoxic drugs (46). Furthermore, elderly patients have more comorbidities than their younger counterparts and thus often take medications that may interfere with the chemotherapeutic drug metabolism. Comorbidities are responsible for an increased mortality in patients even after adjustment for age and stage (6,25,48).

PS is a major prognostic factor of survival in lung cancer patients and is also a guide for the most appropriate treatment of advanced NSCLC. PS, which provides a useful guide in making treatment decisions for younger patients, is often insufficient to assess the overall status of elderly patients (49). Although PS has a prognostic impact in elderly patients, independent of comorbidities it is not a sufficient appraisal of the true situation and comprehensive geriatric assessment (CGA) adds important information (48). Interpretation of PS is markedly more difficult in elderly patients. Atypical depression, malnutrition, dementia and sarcopenia are more common and are possibly underestimated in elderly patients (48). These situations may cause false interpretation of PS. Additionally, certain geriatric occurrences (falls, neglect, abuse and dementia) are also underestimated even by the relatives (49). CGA is a time-consuming procedure and several studies have proposed certain screening tests allowing for selection of patients who require CGA, recommending specific screening tests for all geriatric patients and CGA for selected patients (50–52).

In this study, we demonstrated that age is one of the major prognostic factors affecting survival in lung cancer patients. The rate of mortality was higher in elderly patients; thus, the median survival time of elderly patients was significantly lower compared with that of younger patients in univariate and multivariate analyses. Elderly patients diagnosed with SCLC histology had poorer outcomes compared with those with NSCLC, and older patients with elevated serum LDH levels had shorter survival times compared with those with normal levels. Additionally, elderly patients were found to be more anemic and less responsive to chemotherapy compared with younger patients. However, when older and younger groups were assessed separately, the impact of chemotherapy responsiveness on survival did not differ, suggesting that the disparity of survival between younger and older groups was not associated with chemotherapy response rate.

In terms of criticisms of the present study, we were unable to analyze the mortality as a result of unrelated reasons apart from cancer and their comorbidities. The chemotherapy regimens and toxicity rates were not compared in the present study, as our aim was only to show the significance of age on survival in association with the administration of chemotherapy and other probable prognostic factors. Despite these limitations, this study is important, as data investigating the outcomes of lung cancer in elderly patients and the effect of age on survival in lung cancer is limited.

In conclusion, almost all patients with advanced lung cancer have a poor prognosis. Establishing clear prognostic variables during initial diagnosis may help physicians to decide which patients should be considered for supportive care only, single agent chemotherapy, combination chemotherapy or multimodality treatment options. In this study we demonstrated that the age of patients is one of the major prognostic factors affecting survival in lung cancer patients. Age alone should not preclude these patients from receiving chemotherapy. Treatment decisions should be based on physiological rather than chronological age. Factors that require evaluation in elderly patients include functional status, comorbidity and cognition.

## Figures and Tables

**Table I:** Patient characteristics and distributions of parameters according to patient age.

Parameters	All patients, % (n=110)	<60 years, % (n=57)	≥60 years, % (n=53)	P-value
Patients	100	52	48	
Gender				0.32
Male	91	88	94	
Female	9	12	6	
Histology				0.19
NSCLC	84	81	89	0.26
Adeno	30	25	36	
Squamous	26	23	30	
Other (unclassified)	28	33	23	
SCLC	16	19	11	
Stage of disease				
NSCLC				0.41
Local (stage I+II)	6	4	11	
Locally advanced (stage III)	33	44	34	
Metastatic (stage IV)	45	52	55	
SCLC				0.40
Limited	5	36	17	
Extensive	11	64	83	
Serum hemoglobin level				**0.02**
Low (<11.9 g/dl)	29	19	39	
Normal (≥12 g/dl)	71	81	61	
Serum WBC count				0.20
Normal (<10,999)	77	72	82	
Elevated (≥11,000)	23	28	18	
Serum platelet count				0.65
Normal (<349,000)	65	63	67	
Elevated (≥350,000)	35	37	33	
Erythrocyte sedimentation rate (/h)				0.14
Normal (<40)	45	38	53	
Elevated (≥41)	55	62	47	
Serum LDH level				1.00
Normal (<449 U/l)	75	75	75	
Elevated (≥450 U/l)	25	25	25	
Response to chemotherapy				**0.01**
Yes	47	68	37	
No	53	32	63	
Final status				**0.01**
Alive	72	83	60	
Succumbed	28	17	40	

Significant P-values (<0.05) are highlighted in bold. NSCLC, non-small cell lung cancer; SCLC, small cell lung cancer; WBC, white blood cell; LDH, lactate dehydrogenase.

**Table II:** Univariate and multivariate analyses of survival and clinical and laboratory variables.

Variables	Univariate analysis P-value	Multivariate analysis P-value
Age (<60 vs. ≥60 years)	**0.009**	**0.023**
Gender (female vs. male)	0.415	0.690
Histology (NSCLC vs. SCLC)	0.446	0.104
Stage of disease (non-metastatic vs. metastatic)	0.109	**0.053**
LDH (<450 vs. ≥450 IU/l)	**0.001**	0.263
Hemoglobin (<12 vs. ≥12 g/dl)	0.412	0.158
Leucocyte (<11,000 vs. ≥11,000/mm^3^)	0.829	**0.023**
Platelet (<350,000 vs. ≥350,000/mm^3^)	0.763	0.105
Sedimentation (<40 vs. ≥40 mm/h)	0.524	0.116
Response to chemotherapy (yes vs. no)	**<0.001**	**0.004**

Significant P-values (<0.05) are highlighted in bold. NSCLC, non-small-cell lung cancer; SCLC, small-cell lung cancer; LDH, lactate dehydrogenase.

**Table III:** Survival estimates of patients analyzed according to age.

	Age <60 years		Age ≥60 years	
		
Variables	Median OS (weeks)	P-value	Median OS (weeks)	P-value
Gender (female vs. male)	49.7/53.8	0.923	37.8/49.1	0.465
Histology (NSCLC vs. SCLC)	57.0/NR	0.848	49.1/7.2	**0.009**
Histology subtype (adenocarcinoma vs. other)	49.7/57.0	0.850	62.1/30.8	0.072
Stage of NSCLC (non-metastatic vs. metastatic)	57.0/41.8	**0.035**	62.1/29.8	0.192
LDH (<450 vs. ≥450 IU/l)	NR/50.5	**0.004**	62.1/20.0	**0.042**
Hemoglobin (<12 vs. ≥12 g/dl)	NR/57.0	0.553	NR/37.8	0.689
WBC (<11,000 vs. ≥11,000/mm^3^)	57.0/NR	0.554	49.1/37.8	0.660
Platelet (<350,000 vs. ≥350,000/mm^3^)	NR/57.0	0.493	49.1/NR	0.230
Sedimentation (<40 vs. ≥40 mm/h)	NR/NR	0.560	62.1/20.1	0.226
Response to chemotherapy (yes vs. no)	NR/49.7	0.817	NR/20.1	0.378

Significant P-values (<0.05) are highlighted in bold. NR, not reached; OS, overall survival; NSCLC, non-small cell lung cancer; SCLC, small cell lung cancer; LDH, lactate dehydrogenase.
